# Adult‐onset Still’s disease accompanying noninfective endocarditis

**DOI:** 10.1002/jgf2.339

**Published:** 2020-06-15

**Authors:** Kosuke Oka, Yokota Yuya, Miho Yasuda, Fumio Otsuka

**Affiliations:** ^1^ Department of General Medicine Okayama University Graduate School of Medicine, Dentistry and Pharmaceutical Sciences Okayama Japan

**Keywords:** adult‐onset Still's disease (AOSD), fever of unknown origin (FUO), noninfective endocarditis

## Abstract

Adult‐onset Still's disease can develop valve lesions. Since AOSD may complicate valvular lesion, differentiation of endocarditis in patients with AOSD is required.

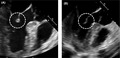

A 42‐year‐old man complained of prolonged fever accompanying a sore throat and bilateral knee joint pain. Laboratory examination revealed an increased white blood cell count (20 510/µL; 83% neutrophils), elevation of CRP (12.6 mg/dL) and transaminases (AST: 41 U/L, ALT: 48 U/L) and moderately increased serum ferritin (657 ng/mL), but blood culture was negative. Based on Yamaguchi's criteria, the patient was diagnosed as having adult‐onset Still's disease (AOSD).^1^ Moreover, since transesophageal echocardiography revealed vegetation on the mitral valve (Figure 1A), ceftriaxone was administered for infectious endocarditis. However, since fever and laboratory findings of inflammatory reaction persisted, oral administration of prednisolone (50 mg) was commenced, and fever, laboratory abnormalities, and the valvular vegetation rapidly improved (Figure 1B). (Written informed consent was obtained from the patient for publication of this case report and accompanying images.)

**FIGURE 1:**
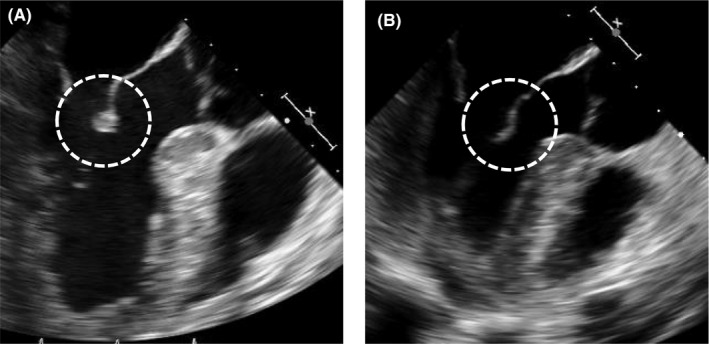
The pictures show mitral valve lesion on transesophageal echocardiography (circle). A, shows before treatment, and (B) shows after treatment

Endocarditis in AOSD has been reported to be due to fibrinoid necrosis with lymphohistiocytic infiltration.^2^ Since AOSD may complicate valvular lesion, differentiation of endocarditis in patients with AOSD is required for the early stage.

## CONFLICT OF INTEREST

The authors have stated explicitly that there are no conflicts of interest in connection with this article.

## INFORMED CONSENT

Written informed consent was obtained from the patient for publication of this case report and accompanying images.
